# Epidemiology and clinical features of imported malaria: a 14-year retrospective single-centre descriptive study in Prague, Czech Republic

**DOI:** 10.1186/s12936-022-04282-8

**Published:** 2022-09-06

**Authors:** Milan Trojánek, Vyacheslav Grebenyuk, Lenka Richterová, Ivana Zicklerová, Eva Nohýnková, Zdenka Manďáková, Jakub Kantor, Hana Roháčová, František Stejskal

**Affiliations:** 1 Department of Infectious Diseases, 2nd Faculty of Medicine, Charles University, Budínova 2, 180 81 Prague, Czech Republic; 2 Department of Infectious Diseases, University Hospital Bulovka, Budínova 2, 180 81 Prague, Czech Republic; 3 Department of Infectious Diseases, Institute for Postgraduate Medical Education, Prague, Czech Republic; 4 Department of Clinical Microbiology, University Hospital Bulovka, Budínova 2, 180 81 Prague, Czech Republic; 5 National Reference Laboratory for the Diagnosis of Tropical Parasitic Infections, Budínova 2, 180 81 Prague, Czech Republic; 6 Department of Infectious and Tropical Diseases, 1st Faculty of Medicine, Charles University, Budínova 2, 180 81 Prague, Czech Republic; 7 Department of Microbiology of the 3rd Faculty of Medicine, Charles University, University Hospital Královské Vinohrady, National Institute of Public Health, Šrobárova 50, 100 34, Prague, Czech Republic; 8 Institute of Immunology and Microbiology, 1st Faculty of Medicine, Charles University, Studničkova 7, 128 00 Prague, Czech Republic; 9 Department of Epidemiology of Infectious Diseases, National Institute of Public Health, Šrobárova, Prague, Czech Republic; 10 Department of Infectious Diseases, Regional Hospital Liberec, Husova 10, 460 63 Liberec, Czech Republic

**Keywords:** Malaria, Imported malaria, Travelers, Travel medicine, Plasmodium, Antimalarials

## Abstract

**Background:**

Malaria represents one of the most important imported tropical infectious diseases in European travellers. The objective of the study was to identify changes in the epidemiological features of imported malaria and to analyse the clinical findings and outcomes of imported malaria.

**Methods:**

This single-centre descriptive study retrospectively analysed the medical records of all imported malaria cases in travellers treated at the Department of Infectious Diseases of University Hospital Bulovka in Prague from 2006 to 2019.

**Results:**

The study included 203 patients with a median age of 37 years (IQR 30–48) and a male to female ratio of 3.72:1. *Plasmodium falciparum* was the predominant species (149/203), and its proportion significantly increased from 35/60 cases (58.3%) in 2006–2011 to 69/80 (86.3%) in 2016–2019 (p < 0.001). In contrast, the incidence of *Plasmodium vivax* malaria decreased from 19/60 cases (31.7%) in 2006–2011 to 5/80 (6.3%) in 2016–2019 (p < 0.001). Malaria was imported from sub-Saharan Africa in 161/203 cases (79.3%). The proportion of travellers from Southeast and South Asia decreased from 16/60 (26.7%) and 6/60 (10.0%) in 2006–2011 to 2/80 (2.5%) and no cases (0.0%) in 2016–2019, respectively (p < 0.001 and p = 0.006). Tourism was the most common reason for travel (82/203), however, the proportion of non-tourists significantly increased over time from 29/60 (48.3%) in 2006–2011 to 55/80 (68.8%) in 2016–2019, p = 0.015. Severe malaria developed in 32/203 (15.8%) patients who were significantly older (p = 0.013) and whose treatment was delayed (p < 0.001). Two lethal outcomes were observed during the study period.

**Conclusions:**

This study demonstrated a significant increase in *P. falciparum* malaria, which frequently resulted in severe disease, especially in older patients and those with delayed treatment initiation. The rising proportion of imported malaria in non-tourists, including business travellers and those visiting friends and relatives, is another characteristic finding analogous to the trends observed in Western European and North American centres. The described changes in the aetiology and epidemiology of imported malaria may serve to optimize pre-travel consultation practices and improve post-travel diagnostics and medical care.

**Supplementary Information:**

The online version contains supplementary material available at 10.1186/s12936-022-04282-8.

## Background

Malaria represents one of the leading causes of travel-associated morbidity and mortality, particularly in travellers from high-income countries to the developing world. In most of the tropics, malaria remains an endemic disease, with 241 million cases reported by the World Health Organization (WHO) in 2020 [1]. Globally, over 95% of cases are reported in sub-Saharan Africa, where *Plasmodium falciparum* is the predominant species. However, there was a significant decrease in cases reported in all other tropical regions, including Southeast Asia, the Americas, and the Western Pacific region since the beginning of the century. This trend is paralleled by a steady decrease in cases caused by *Plasmodium vivax*, the species that causes a substantial mortality burden outside of Africa and remains the most common species in Latin America [2].

According to the GeoSentinel Surveillance Network report, which comprises over 25,000 patients between 1997 and 2006, malaria was the most common specific etiologic diagnosis found in 21% of returning travellers, who presented with fever as a chief complaint to specialized travel medicine clinics primarily located in Europe and North America [3]. Despite the wide availability of therapeutic and preventive measures (including anti-malarial chemoprophylaxis and mosquito bite prevention), most “western” travellers do not adhere to these measures, and the primary care providers’ general consideration of the disease as a diagnostic possibility remains low [4–9]. The WHO World Malaria Reports describe an increase in the global burden of malaria since 2015, particularly in sub-Saharan Africa. In addition, the ongoing COVID-19 pandemic has negatively affected malaria control programmes, worsening the situation [1, 10]. The number of cases annually reported to the European Centre for Disease Prevention and Control (ECDC) remained at a relatively constant level of over 8000 cases per year from 2015 to 2019 with nearly all cases imported to Europe from endemic areas [11]. The annual incidence of imported malaria in the United States has steadily increased since the 1970s, with over 2000 cases reported in 2017 [9].

In the Czech Republic 249 cases of malaria were diagnosed in the period from January 2012 to December 2019 [12]. More than half of these cases were diagnosed at the University Hospital Bulovka, which remains the country’s largest tertiary care centre for tropical infections.

Most imported malaria cases begin as a non-specific febrile illness and may be misdiagnosed as the “flu” or common cold, particularly by primary care physicians, who rarely encounter cases of the disease [13]. However, if left untreated, malaria may progress to severe disease with a fatal outcome. Particularly in the case of *P. falciparum* malaria, delays in diagnostics and treatment are associated with the development of severe disease [14]. Therefore, to improve outcomes in patients with malaria, it is vital to spread awareness and knowledge of this rare disease among primary care physicians as well as hospitalists.

Considering the significant changes in malaria epidemiology in tropical regions, European travellers may provide valuable data for sentinel surveillance of the disease due to their access to resource-rich medical care. The aim of this study was to describe the epidemiological and clinical characteristics of malaria cases diagnosed at one of the largest centres for travel medicine in Central Europe. The findings of this study may serve to optimize pre-travel consultation practices in all middle- to high-income countries and improve post-travel diagnostics and care.

## Methods

A single-centre retrospective descriptive study included all cases of imported malaria in travellers who were treated at the Department of Infectious Diseases of University Hospital Bulovka in Prague from 2006 to 2019. This department represents an academic tertiary care centre for tropical infections in Prague and the Central Bohemian Region, with a catchment area population of 2.5 million.

## Study subjects

The study included all patients who presented with fever or other symptoms associated with malaria, reported epidemiologically relevant stays in endemic regions, and had laboratory confirmed *Plasmodium* spp. infection.

The presence of *Plasmodium* spp. was confirmed in all cases by microscopic evaluation of thick and thin blood peripheral blood smears by an experienced parasitologist (L.R., I.Z. or E.N.). Immunochromatographic tests (Binax NOW! Malaria) were performed on presentation in acute care settings in some cases, however, subsequent microscopic evaluation of peripheral blood smears was performed in all patients. PCR detection with a separate Malaria Parasite Real-time PCR Kit (Liferiver) for each of the *Plasmodium* spp. was implemented in 2015 and performed in selected patients (including those infected with different *Plasmodium* spp. and those partially treated abroad). The study excluded asymptomatic patients without microscopically detectable parasitaemia.

## Clinical data

Clinical data were retrospectively extracted from hospital electronic medical records. Primary data included age, sex, visited country, length of stay, reason for travel, pre-travel preventive measures, symptoms, clinical and laboratory findings, treatment, and outcomes. For the evaluation of clinical and laboratory findings the following categories were used: severe *P. falciparum* malaria (as defined by WHO and published in Guidelines for the treatment of malaria. Third edition) [15], non-complicated *P. falciparum* malaria (including mixed infections with *P. falciparum*), ad non-falciparum or other malaria (including mixed infections with species other than *P. falciparum*).

## Statistical methods

Continuous variables are presented as arithmetical means with standard deviations or as medians with interquartile ranges according to data distribution. The Student’s t-test and the Mann–Whitney test were used for comparisons of continuous variables between two groups.

Categorical variables are presented as absolute frequencies and proportions and compared using the Fisher’s exact test. A p-value of 0.05 was considered statistically significant. Data were analysed using GraphPad PRISM 8.4.3 for Mac (GraphPad Software, San Diego California USA, www.graphpad.com).

## Results

The study included 203 patients. The median age was 37 years (IQR 30–48), and the male to female ratio (M:F ratio) was 3.72:1. A total of 4 cases (2.0%) were diagnosed in children under 18 years and 8 cases (3.9%) in patients over 65 years of age.

**Fig. 1:**
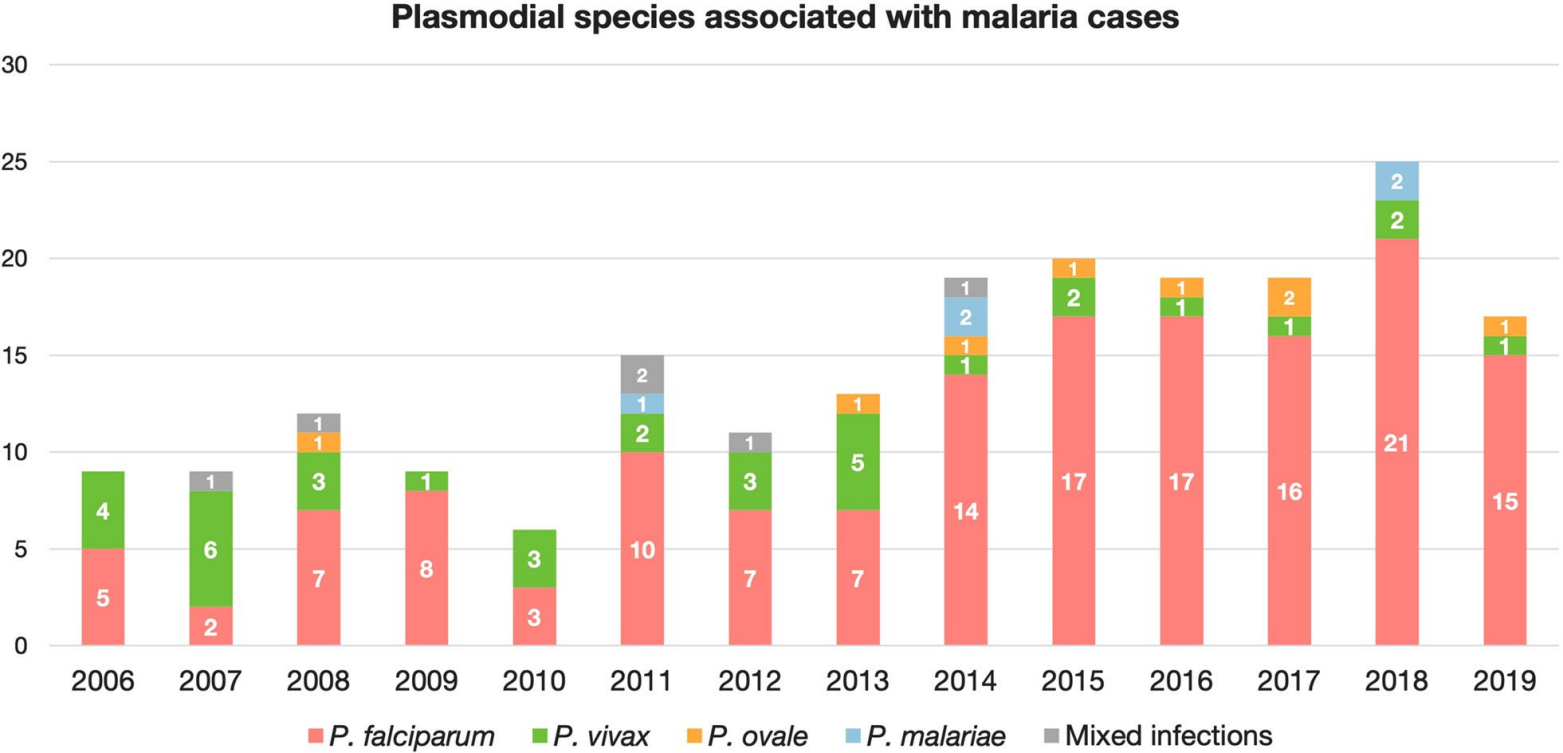
Plasmodial species associated with malaria cases

Malaria was caused by *P. falciparum* in 149 cases (73.4%), *P. vivax* in 35 (17.2%), *Plasmodium ovale* in 8 (3.9%), and *Plasmodium malariae* in 5 (2.5%) patients. Mixed infection was diagnosed in the remaining 6 cases (3.0%): *P. falciparum* and *P. ovale* (4 cases; 2.0%), *P. falciparum* and *P. vivax* (1 case; 0.5%), and *P. ovale* and *P. malariae* (1 case; 0.5%). One case of severe malaria was associated with mixed infection (*P. falciparum* and *P. ovale*). The incidence of different *Plasmodium spp.* is presented in Fig. 1. It is evident, that the proportion of *P. falciparum* malaria significantly increased from 35/60 cases (58.3%) in 2006–2011 to 69/80 (86.3%) in 2016–2019 (p < 0.001), while the proportion of *P. vivax* malaria decreased from 19/60 cases (31.7%) in 2006–2011 to 5/80 (6.3%) in 2016–2019 (p < 0.001). The increasing trend for *P. ovale* malaria was not statistically significant (p = 0.333). Confirmation with real-time PCR was performed in 48/100 patients diagnosed during 2015–2019. There was only one instance of mismatch between microscopic diagnosis and RT-PCR: in 2019, a PCR-confirmed case of *P. ovale* was initially misidentified by microscopy as *P. vivax*.

The reasons for the travel and travel destinations of the patients according to study periods are presented in Table 1. Tourism, as the reason for travel in patients with imported malaria, significantly decreased from 31/60 (51.7) in 2006–2011 to 25/80 (31.3%) in 2016–2019, p = 0.015. Conversely, there was a statistically non-significant trend towards increasing proportions of business travellers (p = 0.065) and those visiting friends and relatives (p = 0.101). In addition, malaria was diagnosed in 18 long-term residents of endemic countries who travelled to Europe and in one internationally adopted child.

**Table 1:** Reason for travel and destination of travellers with malaria

	2006–2011		2012–2015		2016–2019		Total	
	*N*	%	*N*	%	*N*	%	*N*	%
Reason for travel								
Tourism	31	51.7	26	41.3	25	31.3	82	40.4
Business	15	25.0	28	44.4	33	41.3	76	37.4
Visiting friends and relatives	6	10.0	5	7.9	15	18.8	26	12.8
Other	8	13.3	4	6.3	7	8.8	19	9.4
Travel destination								
Sub-Saharan Africa	34	56.7	50	79.4	77	96.3	161	79.3
Southeast Asia	16	26.7	3	4.8	2	2.5	21	10.3
South Asia	6	10.0	4	6.3	0	0.0	10	4.9
Latin America	3	5.0	3	4.8	1	1.3	7	3.4
Other	1	1.7	3	4.8	0	0.0	4	2.0
Total	60	100.0	63	100.0	80	100.0	203	100.0

Most patients returned from sub-Saharan Africa (161/203; 79.3%), followed by Southeast Asia (21/203; 10.3%), and South Asia (10/203; 4.9%). The proportion of travellers returning from sub-Saharan Africa significantly increased from 34/60 (56.7%) in 2006–2011 to 77/80 cases (96.3%) in 2016–2019 (p < 0.001). In contrast, the proportion of travellers returning from Southeast and South Asia decreased from 16/60 (26.7%) and 6/60 (10.0%) in 2006–2011 to 2/80 (2.5%) and no cases (0.0%) in 2016–2019, respectively (p < 0.001 and p = 0.006). The representation of regions and countries most frequently visited by *Plasmodium* species is presented in Table 2. Most *P. falciparum* cases were imported from sub-Saharan Africa (138/149; 92.6%). The most frequent regions of acquisition of P*. vivax* malaria were Southeast Asia and Oceania (15/35; 42.9%). Additionally, there were three cases of *P. falciparum* malaria imported from Oman in 2015, and one case of airport malaria acquired by a worker of an international airport in Lisbon in 2009. *Plasmodium ovale* and *P. malariae* were imported from sub-Saharan Africa in all but one case (12/13; 92.3%).

**Table 2:** Regions and countries of acquisition s and the most frequently visited countries by *Plasmodium*

Region Country	*P. falciparum*		*P. vivax*		*P. ovale*		*P. malariae*		Mixed infections		Total	
	N	%	N	%	N	%	N	%	N	%	N	%
Sub-Saharan Africa	138	92.6	6	17.1	8	100.0	4	80.0	5	83.3	161	79.3
Nigeria	23	15.4	0	0.0	1	12.5	2	40.0	0	0.0	26	12.8
Ghana	17	11.4	0	0.0	3	37.5	0	0.0	1	16.7	21	10.3
Kenya	10	6.7	1	2.9	0	0.0	0	0.0	0	0.0	11	5.4
Central African Republic	10	6.7	0	0.0	0	0.0	0	0.0	1	16.7	11	5.4
DR Congo	8	5.4	0	0.0	0	0.0	0	0.0	1	16.7	9	4.4
Tanzania	7	4.7	0	0.0	0	0.0	0	0.0	0	0.0	7	3.4
Uganda	7	4.7	0	0.0	0	0.0	1	20.0	0	0.0	8	3.9
Cameroon	4	2.7	0	0.0	3	37.5	0	0.0	1	16.7	8	3.9
Ethiopia	3	2.0	4	11.4	0	0.0	0	0.0	0	0.0	7	3.4
Sierra Leone	5	3.4	0	0.0	0	0.0	0	0.0	0	0.0	5	2.5
Gabon	4	2.7	0	0.0	0	0.0	0	0.0	0	0.0	4	2.0
Mali	4	2.7	0	0.0	0	0.0	0	0.0	0	0.0	4	2.0
Zambia	4	2.7	0	0.0	0	0.0	0	0.0	0	0.0	4	2.0
Southeast Asia and Oceania	4	2.7	15	42.9	0	0.0	1	20.0	1	16.7	21	10.3
Indonesia	3	2.0	5	14.3	0	0.0	1	20.0	1	16.7	10	4.9
Papua New Guinea	1	0.7	9	25.7	0	0.0	0	0.0	0	0.0	10	4.9
South Asia	2	1.3	8	22.9	0	0.0	0	0.0	0	0.0	10	4.9
India	1	0.7	6	17.1	0	0.0	0	0.0	0	0.0	7	3.4
Pakistan	1	0.7	1	2.9	0	0.0	0	0.0	0	0.0	2	1.0
Latin America	1	0.7	6	17.1	0	0.0	0	0.0	0	0.0	7	3.4
Peru	0	0.0	4	11.4	0	0.0	0	0.0	0	0.0	4	2.0
Honduras	0	0.0	2	5.7	0	0.0	0	0.0	0	0.0	2	1.0
Haiti	1	0.7	0	0.0	0	0.0	0	0.0	0	0.0	1	0.5
Other	4	2.7	0	0.0	0	0.0	0	0.0	0	0.0	4	2.0
Total	149	100.0	35	100.0	8	100.0	5	100.0	6	100.0	203	100.0

The median length of stay in endemic areas was 24 days (IQR 16–55) after the exception of the long-term residents of endemic countries (n = 29). A total of 31/174 (17.8%) travellers stayed in the endemic regions for less than 2 weeks, 63/174 (36.2%) stayed for 2 to 4 weeks, 39/174 (22.4%) stayed for 4–8 weeks, and 41/174 (23.6%) reported stays longer than 8 weeks. The length of stay was not reported in 10 travellers, and it was irrelevant in long-term residents of endemic countries newly arriving in Europe (19 cases). The duration of stay was significantly longer in business travellers [44 days (IQR 17–71)] and those visiting friends and relatives [30 days (IQR 21–55)] than in tourists [21 days (IQR 16–28)], p < 0.001.

There were 32 patients (15.8%) with severe *P. falciparum* malaria (including one coinfection with *P. ovale*), 122/203 (60.0%) patients with non-complicated *P. falciparum* malaria (coinfection with *P. ovale* in three cases, with *P. vivax* in one case), and 49/203 (24.1%) patients with non-falciparum malaria (dual infection with *P. ovale* and *P. malariae* in a single case). Severe *P. falciparum* malaria represented 7/60 (11.7%) of all malaria cases in 2006–2011, 10/63 (15.9%) in 2012–2015, and 15/80 (18.8%) in 2016–2019, respectively (p = 0.258). No cases of severe non-falciparum malaria were observed.

The most frequently reported chronic co-morbidities were arterial hypertension (21/203; 10.3%), asthma/chronic obstructive pulmonary disease (7/203; 3.4%), hyperlipidaemia (5/203; 2.5%), history of thromboembolic disease (5/203; 2.5%), hyperuricaemia (4/203; 2.0%), depressive disorder (4/203; 2.0%), diabetes mellitus (3/203; 1.5%), and HIV infection (2/203; 1.0%). However, most patients (151/203; 74.4%) reported no co-morbidities.

Anti-malarial chemoprophylaxis was used by three patients (9.4%) with severe *P. falciparum* malaria. However, two of the patients reported using a non-recommended drug (chloroquine and artemisinin), and one patient was non-compliant with atovaquone-proguanil prophylaxis. Fourteen patients (11.5%) with non-complicated *P. falciparum* malaria used anti-malarial chemoprophylaxis: 2 patients reported proper use of mefloquine, 9 patients were non-compliant with chemoprophylaxis, and 3 patients used an inadequate drug. In non-falciparum malaria, chemoprophylaxis was used by 18 patients: 12 patients were compliant with the recommended regimen, and 6 patients were non-compliant. However, most patients with severe malaria [29/32 (90.6%)], non-complicated *P. falciparum* malaria [108/122 (88.5%)], and non-falciparum malaria [31/49 (63.3%)] did not use any chemoprophylaxis (p < 0.001).

Initial and maximal parasitaemias were 12.30% (IQR 6.20–22.00) and 17.28% (IQR 10.44–28.00) in severe *P. falciparum* malaria, 0.39% (IQR 0.06–1.80) and 0.52% (IQR 0.10–2.07) in non-complicated *P. falciparum* malaria, 0.03% (IQR 0.01–0.12) and 0.03% (IQR 0.01–0.16) in non-falciparum malaria (p < 0.001 in both analyses). The median age of patients with severe *P. falciparum*, non-complicated *P. falciparum* and non-falciparum malaria was 44 (IQR 36–56), 35 (IQR 29–48) and 35 years (IQR 30–45), respectively (p = 0.013). The proportions of comorbidities in patients with severe *P. falciparum*, non-complicated *P. falciparum* and non-falciparum malaria were 10/32 (31.3%), 29/122 (23.8%) and 11/49 (22.4%), respectively (p = 0.628). The median duration of fever from symptom onset to clinical examination was 4 days (IQR 3–5) in severe *P. falciparum* malaria, 2 days (IQR 1–3) in non-complicated *P. falciparum* malaria, and 4 days (IQR 2–6) in other cases (p < 0.001). The reported symptoms and clinical signs are presented in Table 3. Only three patients (1.5%) presented without fever. Two of them were visiting of endemic origin and visited their home countries (Nigeria, India). The other was a Czech national who acquired *P. vivax* malaria despite adequate chemoprophylaxis with mefloquine. Patients with severe malaria more often presented with hypotension, tachycardia, tachypnea, dehydration, jaundice, hepatomegaly, prostration, vomiting, and diarrhea than patients with non-complicated *P. falciparum* or non-falciparum malaria. Detailed laboratory findings are presented in Table 4.

**Table 3:** Symptoms and clinical signs in malaria

Symptom or clinical finding	Severe *P. falciparum* malaria		Non-complicated* P. falciparum* malaria		Other malaria		Total		P value
	N	%	N	%	N	%	N	%	
Headache	17/31	54.8	78/121	64.5	27/46	58.7	122	61.6	0.554
Muscle pain	13/31	41.9	47/121	38.8	16/46	34.8	76	38.4	0.807
Joint pain	12/31	38.7	41/121	33.9	19/46	41.3	72	36.4	0.644
Abdominal pain	8/31	25.8	17/121	14.0	7/46	15.2	32	16.2	0.279
Vomiting	19/31	61.3	31/121	25.6	10/46	21.7	60	30.3	< 0.001
Diarrhoea	19/31	61.3	36/121	29.8	6/46	13.0	61	30.8	< 0.001
Hypotension (< 90/60 mmHg)	7/31	22.6	10/121	8.3	1/46	2.2	18	9.1	0.008
Tachycardia (> 100 beats/min)	22/31	71.0	40/121	33.1	9/46	19.6	71	35.9	< 0.001
Tachypnoea (> 20 breaths/min)	10/31	32.3	8/121	6.6	0/46	0.0	18	9.1	< 0.001
Prostration	19/31	61.3	40/121	33.1	2/46	4.3	61	30.8	< 0.001
Dehydration	17/31	54.8	37/121	30.6	6/46	13.0	60	30.3	< 0.001
Jaundice	13/31	41.9	15/121	12.4	4/46	8.7	32	16.2	< 0.001
Hepatomegaly	13/31	41.9	18/121	14.9	5/46	10.9	36	18.2	< 0.001
Splenomegaly	5/31	16.1	11/121	9.1	5/46	10.9	21	10.6	0.524
Total	31/32	96.9	121/122	99.2	46/49	93.9	198/203	97.5

**Table 4:** Laboratory findings in patients with malaria

Laboratory parameter	Severe *P. falciparum* malaria		Non-complicated *P. falciparum* malaria		Non-falciparum malaria		P value
	Median	IQR	Median	IQR	Median	IQR	
WBC (× 10^9^/l)	6.0	4.7–7.9	5.0	3.9–6.4	4.9	3.7–6.3	0.011
ANC (× 10^9^/l)	4.6	3.6–6.8	3.6	2.6–4.7	2.7	1.9–4.1	< 0.001
ALC (× 10^9^/l)	0.8	0.4–1.0	0.7	0.4–1.1	0.8	0.6–1.5	0.047
AMC (× 10^9^/l)	0.4	0.2–0.6	0.4	0.3–0.7	0.6	0.4–0.9	0.002
Hb (g/l)	138	119–149	146	132–155	139	121–145	0.002
HCT (1/1)	0.389	0.329–0.426	0.418	0.385–0.448	0.391	0.361–0.419	< 0.001
PLT (× 10^9^/l)	32	17–70	98	60–145	95	68–131	< 0.001
CRP (mg/l)	167.4	135.5–246.9	76.2	31.7–139.7	74.6	44.1–148.1	< 0.001
Glycemia	6.4	5.2–7.5	6.0	5.3–6.9	5.7	5.1–6.2	0.167
Na (mmol/l)	133	129–136	135	133–138	137	135–139	< 0.001
K (mmol/l)	3.9	3.6–4.3	3.8	3.6–4.0	3.9	3.7–4.2	0.239
Cl (mmol/l)	99	97–104	100	98–103	103	100–105	0.018
BUN (mmol/l)	8.9	5.4–13.4	5.6	4.1–6.5	4.7	3.6–6.0	< 0.001
Cr (μmol/l)	111	90–161	99	82–112	90	82–106	0.005
Bilirubin (mmol/l)	51	33–106	23	17–35	23	17–29	< 0.001
AST (μkat/l)	1.27	0.88–2.26	0.70	0.52–1.05	0.54	0.39–0.86	< 0.001
ALT (μkat/l)	0.93	0.67–1.43	0.66	0.45–1.23	0.65	0.40–1.08	0.063
ALP (μkat/l)	1.41	1.12–1.74	1.24	0.97–1.60	1.20	1.04–1.66	0.231
GGT (μkat/l)	1.21	0.55–1.70	0.86	0.42–1.71	0.63	0.33–2.11	0.466
INR	1.18	1.13–1.35	1.13	1.05–1.22	1.11	1.06–1.23	0.042
APTT	1.29	1.13–1.38	1.10	1.00–1.23	1.22	1.06–1.32	0.002

*WBC* white blood cell count, *ANC* absolute neutrophil count, *ALC* absolute lymphocyte count, *AMC* absolute monocyte count, *Hb* haemoglobin, *HCT* haematocrit, *PLT* platelet count, *CRP* C-reactive protein, *BUN* blood urea nitrogen, *Cr* creatinine, *AST* aspartate aminotransferase, *ALT* alanine aminotransferase, *ALP* alkaline phosphatase, *GGT* gamma-glutamyl transferase, *INR* international normalized ratio, *APTT* activated partial thromboplastin time ratio

A total of 190 patients (93.6%) were hospitalized. Among the 13 cases (6.4%) treated as outpatients, 9 had non-falciparum malaria, and 4 had non-complicated *P. falciparum* malaria. The median length of hospital stay was 11 days (IQR 7–20) in severe *P. falciparum* malaria, 5 days (IQR 4–6) in non-complicated *P. falciparum* malaria, and 4 days (IQR 3–5) in other cases of malaria (p < 0.001). The median length of intensive care in severe *P. falciparum* malaria was 7 days (IQR 1–11).

The treatment of severe *P. falciparum* malaria was initiated in 18/32 (56.3%) patients with parenteral quinine and clindamycin, in 7/32 cases (21.9%) with artemether/lumefantrine or dihydroartemisinin/piperaquine, in 6/32 (18.8%) with mefloquine, and in 1 patient (3.1%) with atovaquone/proguanil. The initial oral therapy was eventually changed to parenteral quinine with clindamycin in 8/14 patients (57.1%). Non-complicated *P. falciparum* malaria was treated in 65/122 (53.3%) patients with artemether/lumefantrine, in 39/122 (32.0%) with mefloquine, in 17/122 cases (13.9%) with atovaquone/proguanil, and in one case with quinine (0.8%). *P. vivax* malaria was treated in 13/33 (39.4%) cases with chloroquine, in 8/33 (24.2%) with atovaquone/proguanil, in 7/33 (21.2%) with mefloquine, and in 5/33 (15.2%) with artemether/lumefantrine; data on treatment were missing in two of the patients. *Plasmodium ovale* malaria (in one case coinfection with *P. malariae*) was treated with chloroquine in 4/9 cases (44.4%), artemether/lumefantrine in 3/9 (33.3%), and mefloquine or atovaquone/proguanil in one patient (11.1%). A total of 33/55 patients with *P. vivax* or *P. ovale* malaria (including coinfections with *P. falciparum* or *P. malariae*) were subsequently treated with primaquine. *P. malariae* infections were treated with artemether/lumefantrine in 2/5 patients (40.0%), atovaquone/proguanil in 2/5 patients (40.0%), and mefloquine in one patient (20.0%).

A total of 15/32 patients (46.9%) with severe *P. falciparum* malaria developed acute respiratory distress syndrome requiring mechanical ventilation in 8/32 patients (25.0%). Other complications of severe *P. falciparum* malaria included septic shock in 12/32 cases (37.5%), altered mental status in 11/32 (34.4%), severe coagulopathy in 10/32 (31.3%), and acute kidney injury requiring urgent renal replacement therapy in 8/32 patients (25.0%). Bacterial superinfection was diagnosed in 14/32 (43.8%) patients with severe *P. falciparum* malaria, in 13/122 (10.7%) with non-severe *P. falciparum* malaria and no patient with non-falciparum malaria (p < 0.001). Relapse or reinfection was diagnosed in 5/35 (14.3%) patients treated with *P. vivax* malaria (2 previously treated with primaquine). Recrudescent malaria occurred in 7/149 (4.7%) cases of *P. falciparum* treated with artemether/lumefantrine and one case of *P. malariae* treated with atovaquone-proguanil. Six cases of *P. falciparum* recrudescence occurred in adult Czech patients, and one was a Beninese child of 5 undergoing international adoption by a Czech family. The median time from initial parasitaemia clearance to *P. falciparum* recrudescence was 17 days (IQR 15–21). All recrudescent cases were uncomplicated. There were two lethal outcomes in patients with severe *P. falciparum* malaria (2/203; 1.0%).

## Discussion

This study reports the epidemiological characteristics of imported malaria diagnosed at a tertiary care centre for infectious diseases in Prague, Czech Republic. Among the main strengths of this study are the relatively high number of included patients and the long study period, which enables the identification of potential changes in the epidemiology of imported malaria in Eastern European settings. However, the observations of this single-centre retrospective analysis require confirmation from more powerful multi-centre studies that should include Central and Eastern European countries. Therefore, this study is also a bid for broader cooperation and partnership among all European centres for imported tropical infectious diseases.

Among the main findings of this study is the steadily increasing incidence of malaria cases imported to the Czech Republic in recent years. Moreover, this rise is primarily attributed to *P. falciparum* malaria, which has the most potential to cause severe disease. These observations can be explained mainly by an increasing proportion of travellers returning from sub-Saharan Africa, which accounted for nearly all malaria cases from 2016 to 2019. Tourism is no longer the most prevalent reason for travel in recent years, and there is an increasing trend in the proportions of the other types of travellers. Travellers for business and visiting friends and relatives (VFR) tended to travel for longer periods, significantly increasing their exposure to malaria. Other studies have shown that non-tourism reasons for travel are associated with lower adherence to anti-malarial chemoprophylaxis [6]. VFR travellers more often visit remote rural areas and use less mosquito bite prevention. Modern pre-travel advice practices should reflect these essential changes in the profile of travellers to malaria-endemic areas.

The low adherence rates to anti-malarials in this study are troubling but not surprising. At least in part, this may be explained by selection bias, as the study only evaluated patients who acquired malaria during their travel, thus lacking the denominator. However, insufficient adherence to anti-malarial chemoprophylaxis is a global problem described in several studies [6, 16]. In addition, this study assessed the adequacy of the patients' drug regimens used for anti-malarial prophylaxis. Not surprisingly, there was a substantial proportion of travellers who reported taking anti-malarial chemoprophylaxis were either taking a non-recommended drug or did not complete their course. These data are particularly of value since such patients may be falsely reported as adherent to anti-malarial chemoprophylaxis in other studies, which only assess binary data (i.e., "yes/no") collected via questionnaires or national reporting systems. Therefore, pre-travel clinics and preventive healthcare providers should be aware of the current recommendations for anti-malarial chemoprophylaxis alongside the trends in malaria resistance patterns to assist patients in selecting the optimal drug.

The number of studies on the epidemiology of travel-acquired malaria conducted in the Central European region remains low. According to the ECDC, the notification rate of malaria in the Czech Republic has ranged between 3 and 4 cases per 1 million population in recent years. Stępień published a report on the epidemiology of imported malaria in Poland in 2014–2018 and a comparison with previous years [17]. Poland is a country with a population over 3,5 greater than that of the Czech Republic. However, only 141 imported malaria cases were reported during the study period, which accounts for the notification rate of 0.7 cases per 1 million population. Similar figures have been reported for Slovakia and Hungary. However, no detailed epidemiological and clinical data from these countries are available. Imported malaria in Austria is approximately three times as frequent as in the Czech Republic, with a notification rate of 8 to 9 cases per 1 million population annually [11, 16]. Strauss et al. reported epidemiological data on 924 malaria cases imported to Austria between 1990 and 2000. The main findings are similar to this study’s: poor adherence to anti-malarial chemoprophylaxis in international travellers and the increasing proportion of *P. falciparum* infections. Vygen-Bonnet et al. reported a sharp increase in imported malaria cases had been documented in Germany in recent years, reflecting both the increasing number of newly arriving refugees and a rising trend in malaria acquisition by native Germans traveling to malaria-endemic countries [18]. Particularly of note is the growing proportion of non-touristic reasons for travel (including work, education, training, and VFR). A similar trend was observed in this study, albeit to a smaller extent. Considering ongoing globalization and increasing numbers of people, who work and live abroad, a similar development may be expected in the Czech Republic. High proportions of imported malaria in VFR and business travellers are particularly characteristic of Western European and Northern American centres [19–23].

The initial signs and symptoms of patients with malaria are notoriously non-specific. Notably, both patients with non-complicated and severe *P. falciparum* malaria tended to present more commonly with gastrointestinal symptoms, including vomiting, diarrhea, and abdominal pain, than patients with non-falciparum malaria. The exact prevalence of diarrhoea and other GI symptoms in malaria remains unclear. However, an association with higher parasitaemia has been suggested by other studies [24, 25]. The most frequent complications of severe malaria included acute respiratory distress syndrome, septic shock, altered mental status, coagulopathy, and acute kidney injury. The overall case fatality ratio was 1%, which is similar to the numbers reported in other European countries [11, 17, 18]. Of note, patients who developed severe diseases tended to be older and present significantly later from symptom onset (median 4 days vs 2 days in non-complicated malaria). Treatment delay has been shown to significantly increase the risk of progression to severe disease in the a recently published meta-analysis [14]. In this study, patients with co-morbidities were not shown to have an increased risk of severe malaria. This is consistent with the literature review by Lüthi et al. [26], which showed that top risk factors associated with malaria deaths in travellers include non-use or incorrect use of anti-malarial chemoprophylaxis, older age, delay in seeking medical care and male sex, but not medical co-morbidities. The impact of gender on the development of severe disease was not directly addressed in this study. Nevertheless, it should be noted that the overall proportion of male patients with malaria considerably exceeded that of female patients. This disparity is indicated in most malaria case series, and the exact reasons are unknown but may be related to behavioural factors (i. e., lower risk perception, non-adherence to preventive measures by male travellers) or sex-related differences in the biological responses to malaria [27–29].

The most frequent laboratory findings included low platelet counts, mildly elevated liver enzymes and markedly elevated CRP. Anaemia is often mentioned among the classical laboratory findings in malaria, but this was not frequently observed in this study. This may be associated with the earlier presentation and lower parasite burdens in this study, as anaemia tends to be a relatively late manifestation, particularly common in patients with severe disease or individuals living in endemic areas suffering frequent reinfections [30–32].

In this study, vivax malaria was the most common non-falciparum malaria and the most important species acquired outside Sub-Saharan Africa (particularly in Asia and Latin America). While infections with *P. vivax* are rarely severe, two crucial clinical considerations should be accounted for. First, while chloroquine is still the drug of choice in most cases of non-falciparum malaria, the resistance of *P. vivax* has been documented and may be on the rise [33]. Therefore, chloroquine resistance should be suspected in travellers returning from Southeast Asia or Oceania (notably, the island of New Guinea), but also in any patient with delayed clearance or early recurrence of parasitaemia. Second, treatment of *P. vivax* infection always requires the eradication of liver hypnozoites with primaquine to prevent relapses of latent infection. There were five cases of recurrent disease caused by *P. vivax* in the present study. However, reinfection could not be reliably excluded in these cases.

Artemisinin-based combinations represent the treatment of choice for *P. falciparum* malaria and chloroquine-resistant non-falciparum malaria and a treatment option for chloroquine-susceptible non-malaria [34]. Infections with unknown species and particularly any severe malaria should be treated as for *P. falciparum* infection, regardless of species diagnosis. Combination therapy is now recommended to reduce the risk of selecting for resistant parasite species, as artemisinin resistance was observed in the regions where monotherapy had been previously used (Cambodia, Thailand, Guyana) [35–37]. In this study, most cases of non-complicated *P. falciparum* malaria were treated with artemether/lumefantrine, the most widely used artemisinin-based combination globally [38]. Artemether typically leads to rapid clinical and parasitological responses several hours after administration and has a very short plasma elimination half-life of about 2 hours. Lumefantrine is lipophilic. Its absorption is limited in the acute phase of illness and may be increased severalfold by taking the drug together with fat-containing food. With its longer half-life, lumefantrine serves to clear any residual parasites after the rapid clearance produced by artemether and protect the partner drug from resistance [39]. However, treatment with artemisinin derivatives (mainly when used in monotherapy) has been associated with frequent treatment failures and recrudescence of symptomatic malaria. The rate of recrudescence observed in this study was 4.7%, which is high compared to other studies [40, 41]. This may be caused by insufficient absorption of artemether-lumefantrine, as recommendations to take the drug with a fatty meal were not followed in all patients, particularly during the earlier periods of the study. However, another explanation may be related to the high proportion of non-immune travellers of European origin in this study. According to the findings of a retrospective study from Sweden late treatment failures may be more common in this population [42]. This problem requires further investigation of possible dose adjustments, extended treatment regimens or alternative ACT combinations. Most cases of treatment failure do not indicate true resistance but rather a form of parasite persistence via non-specific phylogenetically old mechanisms for evasion of toxins [43]. WHO currently recommends that all patients with recurrent infection ≤ 28 days following treatment be treated with an alternative Artemisinin-based combiations known to be effective in the region [34]. The QUINACT trial has shown similar efficacy in re-treating recurrent uncomplicated malaria in African children with the same artemisinin-based combination compared to alternative combinations or quinine plus clindamycin [44]. However, more randomized trials and data for adult patients are needed to corroborate these findings. Artemether/lumefantrine remains the only artemisinin-based combination therapy (ACT) option available in the Czech Republic. Hence the standard practice has been to treat recrudescent *P. falciparum* malaria with a different class drug (i.e., mefloquine or atovaquone/proguanil). Quinine (with clindamycin) is still widely used in Europe to treat severe *P. falciparum* malaria, although randomized trials have shown a clear mortality benefit of intravenous artesunate over quinine [45, 46]. Intravenous artesunate has been designated orphan by the European Medicines Agency. Nevertheless, it has not been granted marketing authorization, and only a few countries have sufficient legal framework available for physicians to prescribe the drug [47, 48].

The main limitations of this single-centre retrospective descriptive study are related to the patient population and data availability. This study included only symptomatic cases diagnosed in acute care settings. Therefore, patients with a clinically inapparent disease or those diagnosed and treated abroad are underrepresented in the study population. In addition, the PCR confirmation and species differentiation were not performed in most cases. As a result, some hidden mixed infections or submicroscopic parasitaemias may have been missed. Finally, the study site lacked the necessary resources for molecular surveillance and investigation of the epidemiologically relevant genetic polymorphisms in *P. falciparum*. A prospective study with data from multiple centres across the country is required to fully describe the epidemiology and clinical spectrum of imported malaria.

## Conclusion

Malaria remains the most important tropical infectious disease in European travellers. Its incidence in some countries may be on the rise, especially with the gradual restoration of international travel during the ebbing phase of the COVID-19 pandemic. Primary care physicians and hospitalists alike are again becoming increasingly likely to encounter a case of malaria. As timely diagnosis and treatment are among the defining factors for favourable patient outcomes, improving general awareness of malaria is crucial for tropical medicine practitioners and researchers. In cooperation with European health authorities, post-travel clinics should strive for equitable accessibility of both oral and intravenous ACTs to ensure the best evidence-based patient management in all regions.

## Supplementary Information

**Additional file 1. **The study's underlying dataset, on which the reported findings are based.

## Data Availability

The dataset supporting the conclusions of this article is included within the article and its supplementary materials.
